# Influenza Vaccinations During the COVID-19 Pandemic — 11 U.S. Jurisdictions, September–December 2020

**DOI:** 10.15585/mmwr.mm7045a3

**Published:** 2021-11-12

**Authors:** Patricia Castro Roman, Karen Kirtland, Elizabeth R. Zell, Nkenge Jones-Jack, Lauren Shaw, Lauren Shrader, Carrie Sprague, Jessica Schultz, Quan Le, Abhinav Nalla, Sydney Kuramoto, Iris Cheng, Mary Woinarowicz, Steve Robison, Shannon Robinson, Kelley Meder, Ashley Murphy, Lynn Gibbs-Scharf, LaTreace Harris, Bhavini Patel Murthy

**Affiliations:** ^1^Immunization Services Division, National Center for Immunization and Respiratory Diseases, CDC; ^2^CDC COVID-19 Rapid Response Team; ^3^Peraton Corporation, Herndon, Virginia; ^4^Stat-Epi Associates, Inc, Ponte Vedra Beach, Florida; ^5^Idaho Department of Health and Welfare; ^6^Iowa Department of Public Health; ^7^Louisiana Department of Health; ^8^Michigan Department of Health and Human Services; ^9^Minnesota Department of Health; ^10^New York City Department of Health and Mental Hygiene, New York; ^11^North Dakota Department of Health; ^12^Oregon Health Authority; ^13^Utah Department of Health; ^14^Washington State Department of Health;^15^Wisconsin Department of Health Services.

Influenza causes considerable morbidity and mortality in the United States. Between 2010 and 2020, an estimated 9–41 million cases resulted in 140,000–710,000 hospitalizations and 12,000–52,000 deaths annually (*1*). As the United States enters the 2021–22 influenza season, the potential impact of influenza illnesses is of concern given that influenza season will again coincide with the ongoing COVID-19 pandemic, which could further strain overburdened health care systems. The Advisory Committee on Immunization Practices (ACIP) recommends routine annual influenza vaccination for the 2021–22 influenza season for all persons aged ≥6 months who have no contraindications (*2*). To assess the potential impact of the COVID-19 pandemic on influenza vaccination coverage, the percentage change between administration of at least 1 dose of influenza vaccine during September–December 2020 was compared with the average administered in the corresponding periods in 2018 and 2019. The data analyzed were reported from 11 U.S. jurisdictions with high-performing state immunization information systems.* Overall, influenza vaccine administration was 9.0% higher in 2020 compared with the average in 2018 and 2019, combined. However, in 2020, the number of influenza vaccine doses administered to children aged 6–23 months and children aged 2–4 years, was 13.9% and 11.9% lower, respectively than the average for each age group in 2018 and 2019. Strategic efforts are needed to ensure high influenza vaccination coverage among all age groups, especially children aged 6 months–4 years who are not yet eligible to receive a COVID-19 vaccine. Administration of influenza vaccine and a COVID-19 vaccine among eligible populations is especially important to reduce the potential strain that influenza and COVID-19 cases could place on health care systems already overburdened by COVID-19.

Influenza vaccination data reported to CDC from 11 study jurisdictions^†^ with high-performing state immunization information systems for persons in the following age groups were analyzed: 6–23 months, and 2–4, 5–12, 13–17, 18–49, 50–64, and ≥65 years. Persons aged ≥6 months with at least 1 dose of influenza vaccine administered between the first week of September and last week of December in 2018, 2019, and 2020, were included in the analysis. The numbers of vaccine doses administered to each age group in 2020 were compared with the average number of reported doses administered during the corresponding weeks in 2018 and 2019. In addition, the percentage change between the number of influenza vaccine doses administered during September–December 2020 and the average administered in the corresponding periods in 2018 and 2019 among persons aged ≥6 months was calculated overall and stratified by age groups. Analyses were conducted with SAS (version 9.4; SAS Institute). This activity was reviewed by CDC and was conducted consistent with applicable federal law and CDC policy.^§^

A total of 16,872,970 influenza vaccine doses were reported by 11 study jurisdictions to state immunization information systems during September–December 2020, compared with an average of 15,513,428 doses reported during the same weeks in 2018 and 2019 (Figure 1), representing an overall increase of 9.0% in influenza doses administered to all age groups compared with 2018 and 2019 (Figure 2). However, the numbers of influenza vaccine doses administered to children aged 6–24 months and children aged 2–4 years were 13.9% and 11.9% lower, respectively than the average numbers administered during September–December of 2018 and 2019. The number of doses administered to children aged 5–12 years was similar in 2018, 2019, and 2020. During September–December 2020, the number of influenza vaccine doses administered increased 12.9% among adolescents aged 13–17 years, the only increase observed among all children, compared with the average during the corresponding period in 2018 and 2019. Influenza doses administered to adults increased in all age groups during September–December 2020, compared with the average during the preceding 2 years: the largest increase (15.3%) was among persons aged 50–64 years, followed by persons aged 18–49 years (14.6%); the smallest increase was among persons aged ≥65 years (9.5%).

**Figure 1:**
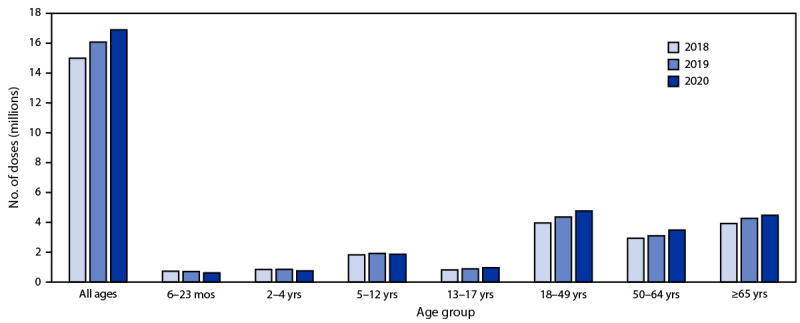
Number of influenza vaccine doses reported to immunization information systems* administered to persons aged ≥6 months during 2020 compared with the number of doses administered during the corresponding period in 2018 and 2019 — 11 U.S. jurisdictions,^†^ September–December 2018, 2019, and 2020 * Vaccine doses were reported to immunization information systems, which are confidential, computerized, population-based systems that collect and consolidate vaccination data from providers in 64 jurisdictions nationwide and can be used to track administered vaccines and measure vaccination coverage. The 64 jurisdictions include the 50 U.S. states, five U.S. territories (American Samoa, Guam, Northern Mariana Islands, Puerto Rico, and U.S. Virgin Islands), three freely associated states (Federated States of Micronesia, Marshall Islands, and Palau), and six local jurisdictions (Chicago, Illinois; Houston, Texas; New York, New York; Philadelphia, Pennsylvania ;San Antonio, Texas; and Washington, DC). ^†^ Study jurisdictions included Idaho; Iowa; Louisiana; Michigan; Minnesota; New York, New York; North Dakota; Oregon; Utah; Washington; and Wisconsin.

**Figure 2:**
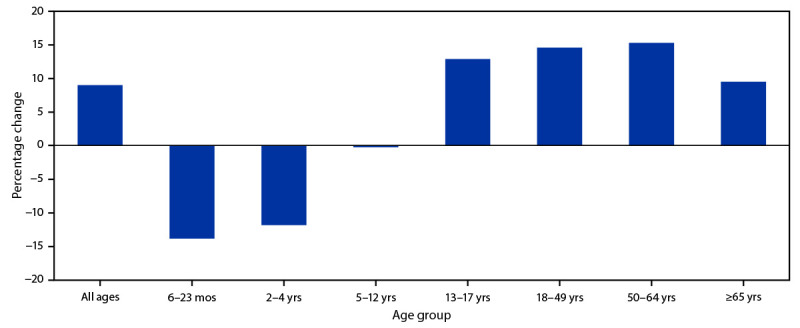
Percentage change* in the number of administered influenza vaccine doses reported to immunization information systems^†^ in persons aged ≥6 months during 2020 compared with the average number of doses administered during the same period in 2018 and 2019 — 11 U.S. jurisdictions,^§^ September–December 2020 * Percentage change in vaccine administration of at least 1 dose of influenza vaccine for September–December 2020 was compared with the corresponding weeks in 2018 and 2019. ^†^ Vaccine doses were reported to immunization information systems, which are confidential, computerized, population-based systems that collect and consolidate vaccination data from providers in 64 jurisdictions nationwide and can be used to track administered vaccines and measure vaccination coverage. The 64 jurisdictions include the 50 U.S. states, five U.S. territories (American Samoa, Guam, Northern Mariana Islands, Puerto Rico, and U.S. Virgin Islands), three freely associated states (Federated States of Micronesia, Marshall Islands, and Palau), and six local jurisdictions (Chicago, Illinois; Houston, Texas; New York, New York; Philadelphia, Pennsylvania; San Antonio, Texas; and Washington, DC). ^§^ Study jurisdictions included Idaho; Iowa; Louisiana; Michigan; Minnesota; New York, New York; North Dakota; Oregon; Utah; Washington; and Wisconsin.

## Discussion

During September–December 2020, the number of influenza vaccine doses administered to persons in 11 reporting U.S. jurisdictions with high-performing immunization information systems increased 9.0% compared with the average for the corresponding period in 2018 and 2019; however, the overall increase was driven largely by increases in doses administered to adolescents and adults. In contrast, the number of influenza vaccine doses administered to children aged 6 months–4 years declined during this period compared with the average during 2018 and 2019. These findings are consistent with those of a 2021 study of influenza vaccination coverage using national survey data (National Immunization Survey-Flu and Behavioral Risk Factor Surveillance System [BRFSS]) that found influenza vaccination coverage was lower among persons aged 6 months–17 years and higher among those aged ≥18 years during the 2020–21 influenza season compared with coverage during 2019–20 (*3*). Whether this finding was attributable to influenza immunization campaigns was unclear; these campaigns emphasized the importance of receiving the annual influenza vaccine to help reduce the spread of influenza viruses. Although the flu vaccine does not protect against COVID-19, influenza vaccination was part of a public health strategy to flatten the curve of respiratory illnesses overall, protect essential workers from influenza, and preserve medical resources for care of COVID-19 patients. 

Influenza activity during the 2020–21 season was unusually low in the United States and worldwide (*5*). Public health measures to limit the spread of SARS-CoV-2, the virus that causes COVID-19, such as wearing face masks, implementing stay-at-home recommendations, promoting good hand hygiene, closing schools, restricting travel, increasing ventilation of indoor spaces, and maintaining physical distancing all likely contributed to the decline in influenza-like illnesses during 2020. Since these COVID-19 mitigation strategies also reduced the spread of influenza viruses, these measures, combined with the transition to hybrid or fully virtual learning, might have led parents to perceive that their children were at lower risk for contracting influenza. Decisions about whether to vaccinate children against influenza might have been influenced by the time of year children received an annual well-child check-up, or by COVID-19–related barriers to health care access, including provider office closures or fear of contracting COVID-19 while getting the influenza vaccine (*5*). Reports have also noted a reduction in routine pediatric vaccine (other than the influenza vaccine) ordering and administration during the COVID-19 pandemic (*6*–*8*), indicating that these barriers might have also discouraged parents and guardians from seeking routine pediatric care for their children, including annual influenza vaccination (*5*).

The findings in this report are subject to at least four limitations. First, findings might not be representative of the entire United States, because only data from 11 jurisdictions were analyzed. Second, data analyzed from immunization information systems might include potentially incomplete vaccination histories that could underestimate vaccine administration in the current analysis. Third, the change in the number of administered doses reported likely overestimates the change in number of persons vaccinated, especially among children aged 6 months–8 years, who require 2 influenza vaccine doses during their first season of vaccination (*2*). Finally, there has been a gradual increase in the number of influenza vaccine doses administered to adults reported via immunization information systems and in the BRFSS survey over the past few years, and the increase in 2020 could be due to continuation of the temporal trend, unrelated to the pandemic.

Given that the 2021–22 influenza season will coincide with the ongoing COVID-19 pandemic, strategic efforts are necessary to ensure high influenza vaccination coverage among all age groups, especially children aged 6 months–4 years, who are not yet eligible to receive a COVID-19 vaccine. ACIP recommends routine annual influenza vaccination for all persons aged ≥6 months who have no contraindications (*2*). With the continued effort to safely keep schools open for in-person learning, and workplaces and businesses resuming in-person activities, CDC recommends that health care providers consider co-administering COVID-19 vaccines with routine vaccines such as influenza (*2*). To address the importance of influenza vaccination during the COVID-19 pandemic, CDC increased the availability of influenza vaccines and conducted targeted communication outreach to groups at higher risk, such as adults aged ≥65 years, young children, pregnant women, and persons with certain chronic conditions. Influenza vaccination in 2020 was part of a comprehensive public health strategy to reduce the prevalence of respiratory illnesses overall, to help protect essential workers from influenza, and preserve medical resources for patients with COVID-19 (*4*). Influenza vaccination among all age groups could help reduce the spread of influenza this fall and winter, and reduce the potential burden that influenza cases could place on health care systems already overburdened by COVID-19.

**Summary**

**What is already known about this topic?**

As the United States enters the 2021–22 influenza season, influenza-associated morbidity and mortality could further strain health care systems already overburdened by the ongoing COVID-19 pandemic.

**What is added by this report?**

During September–December 2020, overall influenza vaccine administration was 9.0% higher than the average during September–December in 2018 and 2019; however, the number of administered doses declined among children aged 6–23 months (13.9%) and 2–4 years (11.9%).

**What are the implications for public health practice?**

Continued strategic efforts are needed to ensure high influenza vaccination coverage among all eligible persons aged ≥6 months, especially children aged ≤4 years.

## Footnotes

* A high-performing immunization information system was defined as a system with vaccine estimates within 10 percentage points of those from the 2018 National Immunization Survey-Child and National Immunization Survey-Teen (https://www.cdc.gov/vaccines/imz-managers/nis/about.html), and which recorded ≥90% of doses administered to persons aged <19 years that were submitted and processed within 30 days of vaccine administration.

† Study jurisdictions included Idaho; Iowa; Louisiana; Michigan; Minnesota; New York, New York; North Dakota; Oregon; Utah; Washington; and Wisconsin. Immunization information systems are confidential, computerized, population-based systems that collect and consolidate vaccination data from providers in 64 jurisdictions nationwide and can be used to track administered vaccines and measure vaccination coverage. The 64 jurisdictions include the 50 U.S. states, five U.S. territories (American Samoa, Guam, Northern Mariana Islands, Puerto Rico, and U.S. Virgin Islands), three freely associated states (Federated States of Micronesia, Marshall Islands, and Palau), and six local jurisdictions (Chicago, Illinois; Houston, Texas; New York, New York; Philadelphia, Pennsylvania; San Antonio, Texas; and Washington, DC).

§ 45 C.F.R. part 46, 21 C.F.R. part 56; 42 U.S.C. Sect. 241(d); 5 U.S.C. Sect. 552a; 44 U.S.C. Sect. 3501 et seq.

## References

[1] CDC. Disease burden of flu. Atlanta, GA: US Department of Health and Human Services, CDC; 2021. Accessed September 1, 2021. https://www.cdc.gov/flu/about/burden/index.html

[2] Grohskopf LA, Alyanak E, Ferdinands JM, Prevention and control of seasonal influenza with vaccines: recommendations of the advisory committee on immunization practices, United States, 2021–22 influenza season. MMWR Recomm Rep 2021;70:1–28. 10.15585/mmwr.rr7005a1 34448800 PMC8407757

[3] CDC. Flu vaccination coverage, United States, 2020–21 influenza season. Atlanta, GA: US Department of Health and Human Services, CDC; 2021. Accessed October 20, 2021. https://www.cdc.gov/flu/fluvaxview/coverage-2021estimates.htm

[4] CDC. 2020–2021 flu season summary. Atlanta, GA: US Department of Health and Human Services, CDC; 2021. Accessed September 10, 2021. https://www.cdc.gov/flu/season/faq-flu-season-2020-2021.htm

[5] Fogel B, Schaefer EW, Hicks SD. Early influenza vaccination rates decline in children during the COVID-19 pandemic. Vaccine 2021;39:4291–5. 10.1016/j.vaccine.2021.06.041 34172330 PMC9756823

[6] Santoli JM, Lindley MC, DeSilva MB, Effects of the COVID-19 pandemic on routine pediatric vaccine ordering and administration—United States, 2020. MMWR Morb Mortal Wkly Rep 2020;69:591–3. 10.15585/mmwr.mm6919e2 32407298

[7] Patel Murthy B, Zell E, Kirtland K, Impact of the COVID-19 pandemic on administration of selected routine childhood and adolescent vaccinations—10 US jurisdictions, March–September 2020. MMWR Morb Mortal Wkly Rep 2021;70:840–5. 10.15585/mmwr.mm7023a2 34111058 PMC8191867

[8] Bramer CA, Kimmins LM, Swanson R, Decline in child vaccination coverage during the COVID-19 pandemic—Michigan care improvement registry, May 2016–May 2020. MMWR Morb Mortal Wkly Rep 2020;69:630–1. 10.15585/mmwr.mm6920e1 32437340

